# A validation study of the Norwegian version of the Health Literacy Questionnaire: A robust nine-dimension factor model

**DOI:** 10.1177/1403494820926428

**Published:** 2020-06-07

**Authors:** Astrid K. Wahl, Åsmund Hermansen, Richard H. Osborne, Marie Hamilton Larsen

**Affiliations:** 1 Department of Interdisciplinary Health Sciences, Institute of Health and Society, University of Oslo, Norway; 2 Department of Social Work, Child Welfare and Social Policy, Faculty of Social Sciences, Oslo Metropolitan University, Norway; 3 Centre for Global Health and Equity, Faculty of Health, Arts and Design, Swinburne University of Technology, Australia

**Keywords:** Confirmatory factor analysis, Health Literacy Questionnaire (HLQ), psoriasis, psychometric testing, validity testing

## Abstract

*Objective:* This study aimed to undertake a rigorous psychometric evaluation of the nine-scale Norwegian version of the Health Literacy Questionnaire (HLQ) based on data from a sample of people with psoriasis. *Methods:* Cross-sectional survey data were collected from 825 adults with psoriasis who previously participated in the Norwegian Climate Heliotherapy programme. To investigate the factorial validity of the Norwegian HLQ, confirmatory factor analyses were carried out using Stata. *Results:* A highly restricted model fit with no cross-loadings or correlated residuals was acceptable for three of the nine scales (‘Feeling understood and supported by health-care providers’, ‘Appraisal of health information’ and ‘Ability to find good health information’). After minor model adjustments of the other scales, one-factor models were acceptable. All scales showed acceptable internal consistency, with Cronbach’s alpha ranging from 0.71 to 0.87. Except for three items, all items had high to acceptable factor loadings. ***Conclusions:* This study of the Norwegian HLQ replicates the original factor structure of the Australian HLQ, indicating the questionnaire has cogent and independent scales with good reliability. Researchers, programme implementers and policymakers could use the Norwegian version of the HLQ with confidence to generate reliable information on health literacy for different purposes.**

## Introduction

Fifteen million deaths attributed to non-communicable diseases (NCDs) occur between the ages of 30 and 69 years, and people from all age groups are vulnerable to the risk factors that contribute to NCDs [1]. The UN Sustainable Development Goal 3.4 states that by 2030, the mortality rate from NCDs needs to be reduced by one third [2–4]. Hence, taking appropriate action is an urgent matter in order to reduce premature mortality related to NCDs. The World Health Organization (WHO) points to health literacy (HL) as an essential factor in the prevention and control of NCDs throughout all stages of life [1], and defines HL as ‘The personal characteristics and social resources needed for individuals and communities to access, understand, appraise and use information and services to make decisions about health. Health literacy includes the capacity to communicate, assert and enact these decisions’ [1,5].

Low HL has been reported to be associated with increased mortality, hospitalisation, lower use of preventive health-care services, poor adherence to described medication, difficulty communicating with health professionals and poorer knowledge about disease processes and self-management skills among people with NCDs. Furthermore, research also suggests that strengths and weaknesses in HL abilities may explain observed health inequalities among people of different races and with different educational attainment [6]. These results are based on the use of different HL measures with variation in content and psychometric robustness across populations and cultures. However, in order to describe the possible HL challenges within and across groups, cultures and populations accurately, and to develop and evaluate the effect of appropriate interventions, it is crucial to have high-quality HL instruments that have undergone modern and robust psychometric testing [7].

While many different HL measurements exist, a main feature of the measurements is whether they are subjective or objective in scope. In objective measurement, people are challenged by standardised test stimuli to measure an underlying trait. In subjective measurement, people self-report their responses to questions about their experiences and skills. Furthermore, HL measures can be characterised as uni- or multidimensional. However, despite promising recent work developing tools in tandem with definitions, the disconnect between definitions and what the tools measure has been a persistent conceptual stumbling block, which has led to several conundrums that will need to be solved for the field to progress in a coherent manner [7].

There is a need for robust HL instruments to be used for health promotion, the prevention of diseases and in disease management in research and practice. The Health Literacy Questionnaire (HLQ) is a generic, subjective, multidimensional instrument (nine domains) measuring HL, developed in Australia by Osborne et al. [6]. The questionnaire was developed using a ‘validity-driven’ instrument development approach and is based on the definition of HL from the WHO noted above. The tool seeks to detect a wide range of HL needs of people in communities and to be used for a variety of purposes, from describing the HL needs of the population in health surveys through to measuring outcomes of public health and clinical interventions designed to improve HL. The HLQ has been translated and adapted for application in many countries and underlies the work done across WHO National Health Literacy Demonstration Projects (NDPHL) across Europe and in other regions [8].

The HLQ has shown psychometric robustness in the original development process (English language) where nine conceptually distinct areas of HL are defined to assess the needs and challenges of a wide range of people and organisations. Across the European versions of the HLQ (France, Denmark, Germany and Slovakia), confirmatory factor analysis (CFA) has been performed and support the initial nine-factor model [9–13]. These diverse studies with disparate patient populations and health-care systems provide strong evidence that the HLQ captures multiple dimensions of HL. The HLQ was translated and culturally adapted into the Norwegian language following a standardised protocol [14]. Data from application of the HLQ in Norway are now available for validity testing, and the present paper describes the psychometric properties of the Norwegian HLQ using robust CFA procedures in a large population of patients with psoriasis.

## Method

### Design and sample

Data from a cross-sectional study of 825 adults with psoriasis who had previously participated in the Norwegian Climate Heliotherapy (CHT) programme was used to investigate the psychometric properties of the HLQ. The CHT is one of the therapeutic options available to Norwegian patients with moderate to severe psoriasis. A three-week multidisciplinary programme is provided in the Canary Islands (located in the Atlantic Ocean at 28°N, 16°W), which includes tailored sunlight ultraviolet (UV)B radiation, physical exercise, group discussions and comprehensive education.

A total of 1275 individuals (all patients participating in the CHT programme from 2011 to 2017) were invited to participate in the study. The data collection period was from March to August 2017. Invitations were sent by post, together with a consent form and the survey. At six weeks following the initial post, a reminder letter was sent. Of the 1275 individuals, 825 (65%) returned a completed survey.

In addition to HLQ data, the present paper draws on self-reported data on sociodemographic information (age, sex, marital status and education) and self-reported health status, co-morbidities, number of CHT treatments and duration of the disease. Reports on associations between these variables and HL, based on the same sample, are published elsewhere [15].

The Regional Committee for Medical Research Ethics for Southern Norway (ID 2016/1745) approved the study. In addition, the administrative leaders of the Section for Climate treatment and the Centre for Privacy and Information Security at Oslo University Hospital approved the study. The study was conducted in accordance with the Declaration of Helsinki.

### The HLQ

The HLQ comprises 44 items representing nine independent HL domains. For further information on the development and the questionnaire, see Osborne et al. [6]. The domains and content descriptors are summarised in Table I.

**Table I.** Overview of the Health Literacy Questionnaire (HLQ): response categories, the nine different domains, number of items for each domain and keywords illustrating a low and high level of health literacy (HL).

Response categories	Domains	Number of items	Low level of HL	High level of HL
1=strongly disagree; 2=disagree; 3=agree; 4=strongly agree	1. Feeling understood and supported by health-care providers	4	• Unable to engage with health-care providers and doctors • No regular health-care provider • Difficulty trusting health-care providers as source of information/advice	• Established relationship with health-care provider • Trust health-care provider to give good advice and information and to assist in decisions about health
	2. Having sufficient information to manage my health	4	• Many gaps in knowledge • Don’t have the information they need	• Feel confident that they have all the information they need
	3. Actively managing my health	4	• Their health is not their responsibility • Don’t engage in health care	• Take responsibility for their own health • Proactively engage in their own care • Make decisions about health • Health a priority
	4. Social support for health	5	• Completely alone and unsupported for health	• All the support they want or need for health
	5. Appraisal of health information	5	• Cannot understand most health information, despite effort	• Able to identify good information and reliable sources of information
1=cannot do; 2=very difficult; 3=quite difficult; 4=quite easy; 5=very eas*y*	6. Ability to engage actively with health-care providers	5	• Passive in approach to health care • Accept information without questions and accept what is offered • No agency in interaction with health-care provider	• Proactive about their health and feel in control in health-care provider relationships • Empowered
	7. Navigating the health-care system	6	• Unable to find help • A limited understanding of what is available and what they are entitled to	• Able to find out about services and supports in order to get their needs met • Able to advocate on their own
	8. Ability to find good health information	5	• Cannot access health information when required • Dependent on others	• Actively use a diverse range of sources to find information and is up to date
	9. Understanding health information well enough to know what to do	5	• Problems understanding written health information or instructions about treatments or medications • Unable to read or write well enough to complete medical forms	• Able to understand all written information • Able to write appropriately on forms when required

Each domain comprises four to six items. The first five domains are scored using response options indicating the level of agreement, ranging from 1=strongly disagree to 4=strongly agree. Four domains report on the capabilities, from 1=cannot do or usually difficult to 5=very easy). The domain scores are calculated as the average of the item scores, with higher scores indicating better HL.

### Data analysis

Analyses were carried out using Stata v16 (StataCorp, College Station, TX). Descriptive analyses were carried out to describe the sample. Further, Cronbach’s alpha was calculated to investigate each scale’s internal consistency.

In order to investigate the factorial validity of the Norwegian version of the HLQ, CFAs were performed. As the structure of the original HLQ has been described previously [6], the factor structure was specified a priori. Consequently, confirmatory analyses were done exclusively. Overall, the amount of missing data per HLQ item was low (1.23–3.13%), with an average of 3.33% of respondents having missing values on one of the nine HLQ scales. CFA models were fitted in Stata v16. For model estimation, maximum likelihood was applied – an estimator that is appropriate for use with ordinal data as is the case with the HLQ.

Model evaluation was based on chi-square tests for model fit and further model fit indices, including the root mean square error of approximation (RMSEA), the comparative fit index (CFI), the Tucker–Lewis index (TLI) and the standardised root mean square residual (SRMR). For model fit to be interpreted as ‘acceptable’, a RMSEA of <0.05 was considered a close fit, while a RMSEA and a SRMR of up to 0.08 were considered acceptable. Comparing the fit of a target model to the fit of an independent or null model, the CFI has a cut-off for good fit CFI of ⩾0.90. A TLI of 0.95 indicates the model of interest improves the fit by 95% relative to the null model, and the cut-off for good fit was sat at TLI ⩾0.95. Furthermore, the correlations of residuals to improve model fit when fitting the nine one-factor models were considered. Correlated residuals <0.2 were considered acceptable when fitting the models [16,17]. Potential model adjustments were based on modification indices as provided in the Stata output using the ‘estat gof, stats (all)’ command.

To obtain a clearer idea of the data and potential problematic items, a full nine-factor model and nine one-factor models were fitted to the data. To test whether modifications, in terms of correlated within-factor residuals, led to significant model improvement, modification indices were obtained using the ‘estat mindices’ command in Stata.

## Results

Sample characteristics are shown in Table II. The total sample consisted of 47.3% women. Age ranged from 21 to 83 years, with a mean age of 53.3 years (*SD*=12.4 years). About 61% of respondents had only formal education of up to 13 years, which is the universal benchmark in Norway.

**Table II.** Sample characteristics for 825 adults with psoriasis who previously participated in the Norwegian Climate Heliotherapy (CHT) programme.

	*n* (%)	*M* (*SD*)	Median (min–max)
Female sex	390 (47.3)		
Age (years)		53.3 (12.4)	
Married/cohabiting	544 (66)		
Primary/secondary school ⩽10 years	94 (11.4)		
Vocational/high school ⩽13 years	407 (49.3)		
College/university <3 years	182 (22.1)		
College/university >3 years	139 (16.9)		
Duration of disease (years)			27 (0–77)
Number of climate heliotherapy treatments			2 (1–39)
Co-morbidities (number)		4.4 (2.5)	
Self-assessed health status (1=poor; 5=excellent)		3.33 (0.92)	
*N*	825		

*SD*: standard deviation.

The median disease duration was 27 years, ranging from 0 to 77 years. Mean self-assessed health was 3.33 on a scale ranging from 1 to 5, with a higher number indicating better health. The mean number of co-morbidities was 4.4 (*SD*=2.5).

Nine one-factor models were fitted to the data, and results are shown in Table III. The results are shown when fitting the Norwegian HLQ as a full nine-factor model with no cross-loadings and no correlated residuals, with χ^2^ (866)=3351.98, *p*<0.001; RMSEA=0.063 (90% confidence interval 0.060–0.065); CFI=0.849; TLI=0.835; SRMR=0.071.

**Table III.** Confirmatory factor analysis of the Norwegian HLQ, full nine-factor model, no cross-loadings, no correlated residuals.

Domain	HLQ scale/item number/question	Standardised factor loading	Standard error
1. Feeling understood and supported by health-care providers	1Q2 I have at least one health-care provider who. . . 1Q8 I have at least one health-care provider I can. . . 1Q17 I have the health-care providers I need. . . 1Q22 I can rely on at least one. . .	0.710 0.744 0.743 0.420	0.026 0.025 0.024 0.033
2. Having sufficient information to manage my health	1Q1 I feel I have good information about health. . . 1Q10 I have enough information to help me deal. . . 1Q14 I am sure I have all the information I. . . 1Q23 I have all the information I need to. . .	0.604 0.796 0.821 0.780	0.026 0.017 0.016 0.018
3. Actively managing my health	1Q6 I spend quite a lot of time actively managing. . . 1Q9 I make plans for what I need to do to be. . . 1Q13 Despite other things in my life, I make time. . . 1Q18 I set my own goals about health and fitness 1Q21 There are things that I do regularly. . .	0.685 0.748 0.695 0.723 0.698	0.023 0.020 0.023 0.021 0.023
4. Social support for health	1Q3 I can get access to several people who. . . 1Q5 When I feel ill, the people around me really. . . 1Q11 If I need help, I have plenty of people I. . . 1Q15 I have at least one person. . . 1Q19 I have strong support from. . .	0.800 0.640 0.747 0.494 0.631	0.019 0.025 0.021 0.031 0.026
5. Appraisal of health information	1Q4 I compare health information from different. . . 1Q7 When I see new information about health, I. . . 1Q12 I always compare health information from. . . 1Q16 I know how to find out if the health. . . 1Q20 I ask health-care providers about the quality. . .	0.733 0.749 0.754 0.516 0.486	0.022 0.021 0.021 0.031 0.032
6. Ability to engage actively with health-care providers	2Q2 Make sure that health-care providers understand. . . 2Q4 Feel able to discuss your health concerns with a. . . 2Q7 Have good discussions about your health. . . 2Q15 Discuss things with health-care providers. . . 2Q20 Ask health-care providers questions to get. . .	0.744 0.767 0.735 0.724 0.778	0.018 0.017 0.018 0.019 0.016
7. Navigating the health-care system	2Q1 Find the right health care 2Q8 Get to see the health-care providers I need to 2Q11 Decide which health-care provider you need. . . 2Q13 Make sure you find the right place to get. . . 2Q16 Find out what health-care services you are. . . 2Q19 Work out what is the best care for you	0.764 0.761 0.713 0.776 0.685 0.690	0.017 0.017 0.020 0.016 0.021 0.021
8. Ability to find good health information	2Q3 Find information about health problems 2Q6 Find health information from several. . . 2Q10 Get information about health so you are. . . 2Q14 Get health information in words you. . . 2Q18 Get health information by yourself	0.726 0.730 0.684 0.642 0.736	0.020 0.020 0.022 0.024 0.019
9. Understand health information well enough to know what to do	2Q5 Confidently fill medical forms in the correct. . . 2Q9 Accurately follow the instructions from. . . 2Q12 Read and understand written health. . . 2Q17 Read and understand all the information on. . . 2Q21 Understand what health-care providers are. . .	0.644 0.533 0.704 0.617 0.705	0.025 0.029 0.023 0.026 0.023

For item content description, see table 4 in Osborne et al.^6^

All factor loadings were high to acceptable (i.e. >0.5; see column ‘Standardised factor loading’ in Table III), except for three items which had somewhat low loading: ‘I can rely on at least one health-care provider’ (0.420) in domain 1 (‘Feel understood and supported by health-care providers’, ‘I have at least one person who can come to medical appointments with me’ (0.494) in domain 4 (‘Social support for health’) and ‘I ask health-care providers about the quality of the health information I find’ (0.486) in domain 5 (‘Appraisal of health information’; Table III). In order to understand these results further, the authors (A.K.W., Å.H. and M.H.L.) compared the original English items (mentioned above) with the Norwegian items, and examined them again with regard to meaning equivalence at the item and phrase level, with reference to the original English item intent. We could not detect any misunderstandings in the Norwegian wordings compared to the English content. One explanation could be that in Norway, it is somewhat uncommon to ask someone other than your GP for advice and guidance about personal health matters. Consequently, this may have resulted in somewhat lower observed factor loadings.

When fitting the one-factor models, correlated residuals were sequentially added to respective models, which improved each model fit significantly. Table IV shows the results of the CFA separately for the nine individual HLQ scales.

**Table IV.** Confirmatory factor analysis and internal consistency (Cronbach’s alpha) of the Norwegian HLQ (one-factor models).

Model	χ²	*p*	RMSEA	CFI	TLI	SRMR	Correlated error	Cronbach’s alpha
**1. Feeling understood and supported by health-care providers**								
Original	4.99	0.082	0.042	0.997	0.990	0.017		0.71
**2. Having sufficient information to manage my health**								
Original	19.83	0.000	0.105	0.986	0.959	0.024		0.83
1Q1 with 1Q10	0.896	0.344	0.000	1.000	1.000	0.005	0.184	
**3. Actively managing my health**								
Original	20.39	0.000	0.062	0.989	0.978	0.020		0.83
1Q9 with 1Q18	8.12	0.087	0.036	0.997	0.993	0.013	0.172	
**4. Social support for health**								
Original	39.03	0.000	0.093	0.972	0.943	0.032		0.80
1Q15 with 1Q19							0.133	
1Q3 with 1Q19							−0.228	
1Q3 with 1Q15	1.37	0.242	0.022	1.000	0.997	0.006	−0.131	
1Q5 with 1Q15							0.104	
**5. Appraisal of health information**								
Original	8.591	0.127	0.030	0.996	0.993	0.020		0.77
**6. Ability to engage actively with health-care providers**								
Original	22.76	0.000	0.067	0.990	0.980	0.020		0.87
2Q15 with 2Q20	5.28	0.259	0.020	0.999	0.998	0.009	0.182	
**7. Navigating the health-care system**								
Original	142.46	0.000	0.136	0.940	0.900	0.045		0.87
2Q16 with 2Q19							0.316	
2Q8 with 2Q19							−0.273	
2Q8 with 2Q13	6.50	0.165	0.028	0.999	0.996	0.009	−0.205	
2Q13 with 2Q19							0.192	
2Q13 with 2Q16							0.141	
**8. Ability to find good health information**								
Original	10.87	0.054	0.038	0.996	0.992	0.015		0.83
**9. Understand health information well enough to know what to do**								
Original	57.84	0.000	0.115	0.948	0.895	0.045		0.78
2Q9 with 2Q21	5.12	0.274	0.019	0.999	0.997	0.012	0.280	

RMSEA: root mean square error of approximation; CFI: comparative fit index; TLI: Tucker–Lewis index; SRMR: standardised root mean square residual.

While model fit without modifications was acceptable for ‘Feeling understood and supported by health-care providers’, ‘Appraisal of health information’ and ‘Ability to find good health information’, one or more correlated residuals were observed in the remaining six scales. Correlated residuals were <0.2 for the majority of the model adjustments, with the exception of one model adjustment (−0.228) in domain 4 (‘Social support for health’), three adjustments (0.316, −0.273 and −0.205) in domain 7 (‘Navigating the health-care system’) and one adjustment (0.280) in domain 9 (‘Understanding health information well enough to know what to do’). After respective model adjustments, the one-factor models were acceptable. All nine HLQ scales showed satisfactory to good internal consistency, with Cronbach’s alpha ranging from 0.71 to 0.87 (see Table IV).

## Discussion

This study of the Norwegian version of the HLQ supports the original Australian nine-dimension HLQ using robust CFA procedures (both full-model and one-model approaches). The model fit (RMSEA and SRMR) for the full nine-factor model is acceptable, although the CFI and TLI is just below 0.85, which is somewhat lower than the original Australian version [6]. However, results showed that after respective model adjustments for some of the scales, the factor models were acceptable. The factor loadings were high to acceptable for almost all items, and the Cronbach’s alpha showed acceptable internal consistency across all scales. Although some of the correlated residuals for model adjustments are just above 0.20, they are small and should not indicate a problem with item-factor loadings. The proportion of non-response to items was small (<3.13%), suggesting that items were well understood and had acceptable content. Ahead of our study, the Norwegian version was translated, applying rigorous linguistic and cultural adaptation methods in order to produce a high-quality Norwegian version [14,18]. Consequently, the Norwegian version of the HLQ appears to be robust and might serve as a good foundation for the measurement of HL in Norwegian populations.

Our results are also in accordance with similar studies from other countries investigating the HLQ structure. Despite differences in samples and statistical programmes/procedures, the Danish, German, Slovakian and French versions of the HLQ all demonstrated comparable results [9–13] with regard to the nine-factor model. Despite some possible overlap between scale 7 (‘Navigating the health-care system’) and scale 8 (‘Ability to find good health information’) in part 2 of the questionnaire, the HLQ appeared relatively robust. The reason for the good fit found across studies is likely a result of the way the HLQ was developed, translated and adapted [6]. The thorough validity-driven development approach included concept mapping workshops and interviews to identify conceptually distinct domains. Questionnaire items were developed directly from consultation data following a strict process aiming to capture the full range of experiences of people currently engaged in health care through to people in the general population. The psychometric analysis included CFA and item response theory. Cognitive interviews were used to ensure that questions were understood as intended. Items were tested in a calibration sample from community health, home care and hospital settings and then in a replication sample comprising recent emergency department attendees [6].

When adapting the questionnaire into a language-specific context, such as Norwegian, the procedure of translation and adaption has been highly standardised across countries, including transparent translation steps, the use of item intents and cognitive interviews [6,14]. The development of the HLQ reported in 2013 gave rise to global interest. In a follow-up study of the psychometric properties of the HLQ including respondents from a diverse range of community-based organisations, the instrument was found to be highly reliable. All HLQ scales were found to be homogenous, and the factor structure of the HLQ was replicated. With a small number of exceptions involving no invariance of factor loadings, strict measurement invariance was established across the participating organisations and sex, language background, age and educational level of respondents [19]. A study evaluating the measurement properties of the HLQ using Rasch analysis among older adults presenting to an emergency department after a fall showed that all nine scales of the HLQ were unidimensional, with good internal consistency [20]. Given this validity-driven approach, the HLQ is likely to be useful in surveys, intervention evaluation and studies of the needs and capabilities of individuals across countries, ages, sex, education and cultures.

Today, the HLQ is used in many studies across countries and health contexts, and gives valuable information on HL from a broader and more in-depth perspective compared to traditional and functional measures of HL [15,21–30]. Globally, directed by the WHO, and in many countries across the world, including Norway, HL has become an important key to solving health challenges in communities and countries. However, to be able to act on HL, policymakers and clinicians need meaningful and valid tools that generate data to identify HL needs, challenges and strengths across settings. Based on the extensive rigorous and ongoing validity testing behind the HLQ, there is mounting evidence that in Norway, the HLQ will provide policymakers, clinicians and researchers with good information for public health and policy decisions. As noted in the introduction, the instrument is useful for both health promotion and disease management settings, since the interpretation of the responses of HLQ can be salutogenic (higher HL is a promotional resource to be strengthened/maintained for health) and pathogenic (lower HL is associated with risks for disease and needs action). Health-care professionals or health workers may use the instrument to explore what HL supports a wide range of the target groups might need.

Validity testing of instruments such as the HLQ is comprehensive and ongoing work, including qualitative and quantitative techniques in order to build evidence. The present study focuses on psychometric properties of the Norwegian version of the HLQ and includes a large number of respondents (*N*=825), the use of robust CFA procedures and a well-developed original questionnaire. A weakness maybe the homogenous sample, including only individuals with psoriasis. While the patient group may not represent the broad Norwegian population, participants had an average of more than four other co-morbidities and may well be similar to common groups with chronic conditions. The response rate was reasonable high (65%), with good variation with regard to sociodemographic characteristics, and participants were situated in different parts of Norway. Although the present study supports the use of the HLQ in Norway, studies in other NCD groups and in the general population are warranted.

### Practice implications

There is mounting evidence that supports the interpretation and use of the HLQ in Western cultures, including Norwegian and other European settings. Our study builds upon growing evidence of the measurement properties of HLQ and that it is a valuable tool for researchers, programme implementers and policymakers to use to explore HL in diverse populations.
